# Color Doppler ultrasonography targeted reconstruction using pedicled perforator flaps—a systematic review and meta-analysis

**DOI:** 10.1007/s00238-018-1435-y

**Published:** 2018-06-29

**Authors:** Rami Mossad Ibrahim, Gudjon Leifur Gunnarsson, Javed Akram, Jens Ahm Sørensen, Jørn Bo Thomsen

**Affiliations:** 1 Department of Plastic Surgery, Odense University Hospital, Odense, Denmark; 2 Plastic Surgery Department, Herlev Hospital, Copenhagen, Denmark; 3 Department of Plastic Surgery, Telemark Hospital, Skien, Norway; 4 Department of Plastic Surgery, Lillebaelt Hospital, Vejle and Odense University Hospital, Odense, Denmark

**Keywords:** CDU, Perforator, Reconstruction, Pedicled, Flaps

## Abstract

**Background:**

Flaps are increasingly popularized in reconstructive surgery and there is need to test and increase their reliability. Color Doppler ultrasound has been stated to be valuable in flap planning. The aim of this study was to conduct a systematic review and meta-analysis of the literature of Color Doppler ultrasound targeted pedicled perforator flaps and provide information on outcomes and complication rates.

**Method:**

A systematic review and meta-analysis were conducted for articles published until April 2017 in PubMed and Embase. We aimed to include randomized clinical trials, meta-analysis, prospective studies, case-control studies, and cohort studies written in English. We included studies where CDU was used to identify the perforator(s) prior to surgery. We evaluated the quality of the included studies using checklists recommended by the Cochrane group.

**Results:**

From the initial 219 studies, only 12 studies using Color Doppler targeted pedicled perforator flaps in 252 cases met the inclusion and exclusion criteria. Eleven of these were case series and one a prospective study. The incidence of major complications was 8% (21/252) and minor complications was 14%, comprising of mostly necrosis 8% (24/252) and venous congestion 8% (21/252).

**Conclusions:**

The reconstructive success rate following pedicled perforator flap reconstruction targeted by CDU appears to be high and the procedure provides a wide scope of applications and margin of safety. It is evident that the risk of venous congestion is 11 times greater in the lower extremities than the truncus, a finding that needs further attention in future studies.

Level of Evidence: Level IV, risk/prognostic study

## Introduction

Knowledge about perforator anatomy has led to an increased use of pedicled perforator flaps for reconstruction throughout the body [1]. Pedicled perforator flaps allow the surgeon to relocate local tissue and facilitate a simple reorganization, which enables an optimal cosmetic and functional reconstructive outcome. They provide a fast and simple, single-stage solution and offer an alternative to microsurgery or skin graft [2].

Handheld Doppler and color Doppler ultrasonography (CDU) have been shown to be useful to identify perforators and aid in the planning of flap reconstructions [2].

CDU provides additional visual information about available soft tissue, vessel flow patterns, vessel course through the soft tissue as well as perforator size and location. However, the use of CDU is not widely reported for use in the planning of pedicled perforator flaps reconstruction [3].

The aim of this systematic review was to evaluate the existing literature regarding color Doppler ultrasonography used to identify perforators for pedicled perforator flap reconstruction and evaluate the associated risk of major and minor complications.

## Methods

This systematic review was conducted according to the recommendations outlined in the Cochrane Handbook for reviews [4] and the PRISMA statement (Preferred Reporting Items for Systematic Reviews and Meta-Analyses) [5].

### Literature search

We performed a literature review regarding the use of CDU in the preoperative assessment and planning of pedicled perforator flap reconstruction in April 2017 in the PubMed and EMBASE databases, using the search string:

((CDU OR color doppler ultrasound OR color doppler ultrasonography OR ultrasound)) AND (perforator flap) AND (pedicled)

**Fig. 1:**
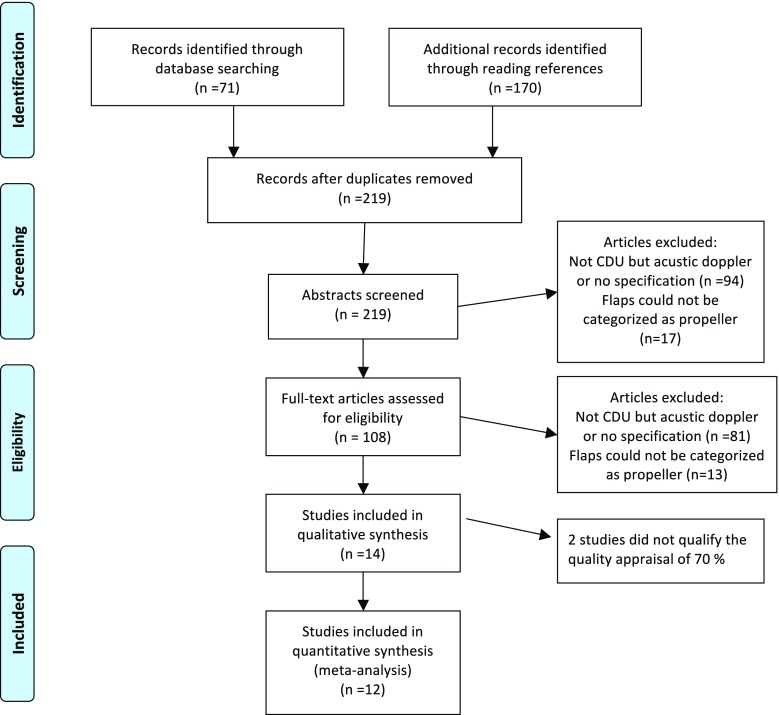
Prisma flow diagram of the number of records identified, included, and excluded, and the reasons for exclusions

The search yielded 71 studies. The retrieved articles were reviewed and their bibliographies were scanned for publications relevant for this review (Fig. 1).

### Inclusion criteria

We planned to include randomized clinical trials, meta-analysis, prospective studies, case-control studies, and cohort studies written in English. We only included studies where CDU was used to identify the perforator(s) prior to surgery using pedicled perforator flaps for reconstruction of local defects.

**Fig. 2:**
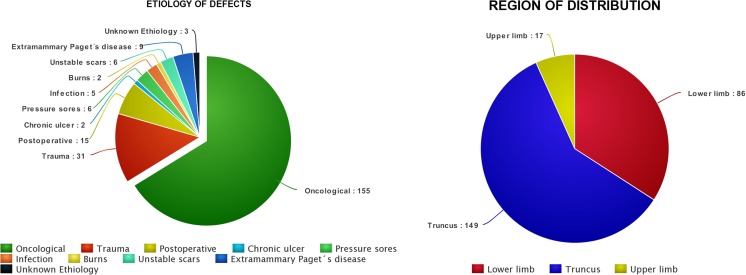
Figures showing distribution of etiologies (left), distribution of flap type (right)

We recorded demographics, etiology and location of the defect, the type of pedicled perforator flap used, size of the flap, and arc of rotation. We also recorded minor complications defined by conservative treatment or by use of local anesthesia and major complications defined by return to the operating theater in general anesthesia (Table 1, Fig. 2).

**Table 1:** Characteristics of the included studies including complications

First authors, year, location (reference)	Information regarding CDU	Number of flaps	Type/location of flaps	Arc of rotation	Size of flap	Complications	
						Major	Minor
Zang 2015	Doppler ultrasound probe was used to identify at least two large perforators adjacent to the defects at different intercostal spaces. Then, the one with the most prominent Doppler signals was selected as the preferred supply for the flap.	9	Truncus: 1 DICAP 4 DLICAP 3 LICAP 1 AICAP	4 = 150° 5 = 180°	6 × 6 cm–30 × 20 cm	2 flaps partial necrosis that needed repair with AICAP propeller flaps	1 flap marginal necrosis (2 cm)
Hamdi 2015	No information	31	Truncus: 18 TDAP 10 MS-LD 3 ICAP	No information	Length: 16–25 cm Width: 6–10 cm Average size: 20 × 8 cm	Partial flap necrosis occurred in 2 cases. Both necessitated a surgical debridement and direct closure.	A small skin slough occurred in one TAP flap that healed spontaneously. Minor wound dehiscence in the donor site occurred in 2 patients (6%). 4 flaps experienced venous congestion.
Gravannis 2006	All measurements were performed by the same observer using an ATL 3500 (Philips, Bothell, WA, USA) ultrasound machine equipped with a 5-MHz and 7.5-MHz linear color Doppler transducer.	11	Truncus: 4 ALT Lower limb: 7ALT	180°	Length: 15–22 cm Width: 8–11 cm	All flaps survived completely, resulting in excellent functional and esthetic results.	1 patient with slightly limited range of motion. 2 patients with muscle weakness that resolved after 6 months.
Innocenti 2015	No information	14	Upper limb: 14 radial forearm flap	180°	No information	1 case used for thenar eminence resurfacing developed necrosis and needed salvage with kite flap.	2 patients with venous congestion that relieved spontaneously, 1 patient with epidermolysis.
Tos 2011	No information	22	Lower limb: 6 peroneal artery perforator 13 posterior tibial artery perforator 1 genicular artery perforator 1 lateral circumflex artery perforator 1 deep femoral artery perforator	80°–180°	3 × 5 cm–12 × 25cm	1 flap necrosis of 50% treated with skin graft, 1 flap necrosis 80%, and 1 diabetic patient with epidermolysis that needed skin graft	5 patients had a limited superficial epidermolysis for venous congestion that resolved spontaneously. 3 patients showed transient venous congestion of the flap. Prolonged leg edema with spontaneous resolution was observed in a patient with a large propeller flap covering an Achilles tendon allograft.
Pignatti 2007	No information	6	Lower limb: No detailed description on perforator origin	2 × 90°, 2 × 135°, and 2 × 180°	8 × 9 cm–25 × 12cm	None	One flap with small superficial necrosis of the tip, due to venous congestion because of inclusion in the design of an already scarred tissue at the tip of the flap. One other patient with a transient venous congestion was observed that resolved spontaneously.
Gunnarson 2015	Used a BK Medical color Doppler ultrasonographer with a 10–12 MHz linear transducer. The settings were set for small peripheral vessels and low flow velocity to enable detection of flow in the perforators.	17	12 Upper limb 13 Lower limb 9 Truncus No detailed description on perforator origin	21 × 90°–13 × 180°	1.5 × 3 cm–12 × 22 cm	None	Minor complications were registered in 4/17 (24%); marginal necrosis was significant in 4 cases, however never more than 10% of the total flap size.
Dong 2014	No information	20	Lower limb: 15 peroneal artery perforator 5 posterior tibia artery perforator flap	180°	5 cm × 11 cm–12 cm × 28 cm	None	1 patient had a venous crisis in the 24 h postoperatively, which responded to removal of some of the sutures and drainage of blood.
Jacobs 2015	No information	99	Truncus: 99 TAP	No information	7 × 21cm–11 × 37cm	1 hematoma, 2 venous congestion that needed surgical intervention and partial flap necrosis in 7.	14 patients with minor complications not described further.
Moscatiello 2007	No information	6	Lower limb: 6 ALT perforator	180°	No information	1 flap with partial necrosis > 20% and the defect was covered with medial gastrocnemius flap	None
Umemoto 2009	No information	4	Lower limb: 4 sural artery perforator	No information	4 × 6 cm–10 × 20 cm	None	None
Jakubietz 2014	No information	7	Lower limb: 3 posterior tibial artery perforator 1 anterior tibial artery perforator 3 peroneal artery perforator	90°–180°	4 × 7 cm–5 × 24 cm	In 1 patient, a noninsulin-dependent diabetic smoker, tip necrosis became apparent 4 days postoperatively. Debridement of the distal part of the flap, negative pressure therapy, and skin graft. In 1 patient with peripheral vascular disease developed superficial epidermolysis in both tips of flap, which also required skin grafting 10 days after the first surgery.	None

*DICAP* dorsal intercostal artery perforator, *DLICAP* dorsolateral intercostal artery perforator, *LICAP* lateral intercostal artery perforator, *AICAP* anterior intercostal artery perforator, *TDAP/TAP* thoracodorsal artery perforator, *MS-LD* muscle sparring latissimus dorsi, *ICAP* intercostal artery perforator, *ALT* anterolateral thigh

### Quality assessment of the studies

We evaluated the quality of the included studies using checklists recommended by the Cochrane group [6]. The Carmen Mogas checklist was used to evaluate the quality of case series and prospective chart reviews [7]. Six questions were not applicable and thus omitted (Table 2).

**Table 2:** Critical appraisal of included studies using the Institute of Health Economics Quality Appraisal tool

Article authors	Quality appraisal								
	Study objective^a^	Study design^b^	Study population^c^	Intervention^d^	Outcome measures^e^	Statistical analysis^f^	Results and conclusions^g^	Competing interests and sources of support^h^	Total
Zang 2015	1	0	1	1	1	1	4	1	10
Hamdi 2015	1	0	1	1	1	1	3	0	8
Gravannis 2006	1	0	1	1	1	1	3	0	8
Innocenti 2015	0	0	1	1	1	1	4	1	9
Tos 2011	1	0	1	1	1	1	4	1	10
Gunnarson 2014	1	0	1	1	1	1	3	1	9
Dong 2014	1	0	1	1	1	1	4	1	10
Jacobs 2015	1	0	1	1	1	1	4	1	10
Moscatiello 2007	1	0	1	1	1	1	4	0	9
Umemoto 2009	1	0	1	1	1	1	4	0	9
Pignatti	1	0	1	1	1	1	3	0	8
Jakubietz 2014	1	0	1	1	1	1	4	1	10

^a^Maximum score 1, ^b^Maximum score 2, ^c^Maximum score 1, ^d^Maximum score 1, ^e^Maximum score 2, ^f^Maximum score 1, ^g^Maximum score 4, ^h^Maximum score 1; studies with total scores of ≥ 70% are considered to be of acceptable quality (19)

### Statistical analysis

We conducted a meta-analysis for outcomes of complications; any necrosis, venous congestion, and flap loss. We calculated proportions with a 95% confidence interval (CI) based on a random-effects model due to the heterogeneous nature of the studies [8]. The heterogeneity was investigated using chi-squared and the *I*^2^ statistics. All statistical analyses were conducted using Stata/IC 14.0 (StataCorp LP) and supervised by a statistician at Odense University Hospital.

## Results

We evaluated 71 studies from the research databases and 170 by assessing the reference lists (Fig. 1). We included 12 studies, 11 case series/retrospective chart reviews, and one prospective study. The studies described 252 CDU targeted pedicled perforator flaps used for reconstruction in 246 patients; 72 male, 153 female, and 21 gender not described [3, 9–19]. The mean age was 53 (36–79) years. The defects needing reconstruction were located in the upper limb in 17/252 cases (7%), lower limb 86/252 (34%), and trunk 149/252 (59%) (Fig. 2). The reconstructive goal was achieved in 247/252 (98%) cases. The size of the flaps used for reconstruction was reported in 240/252 (95%) cases and varied from 4.5 to 600 cm^2^. In the upper limb, the size of the flaps varied between 4,5 and 136 cm^2^, 40 and 600 cm^2^ in the torso, and 15 and 400 cm^2^ in the lower limb. The main indication for reconstruction was an oncological defect 155/252 (61%), post-traumatic 31/252 (12%), and other surgery 15/252 (6%) (Fig. 2).

**Fig. 3:**
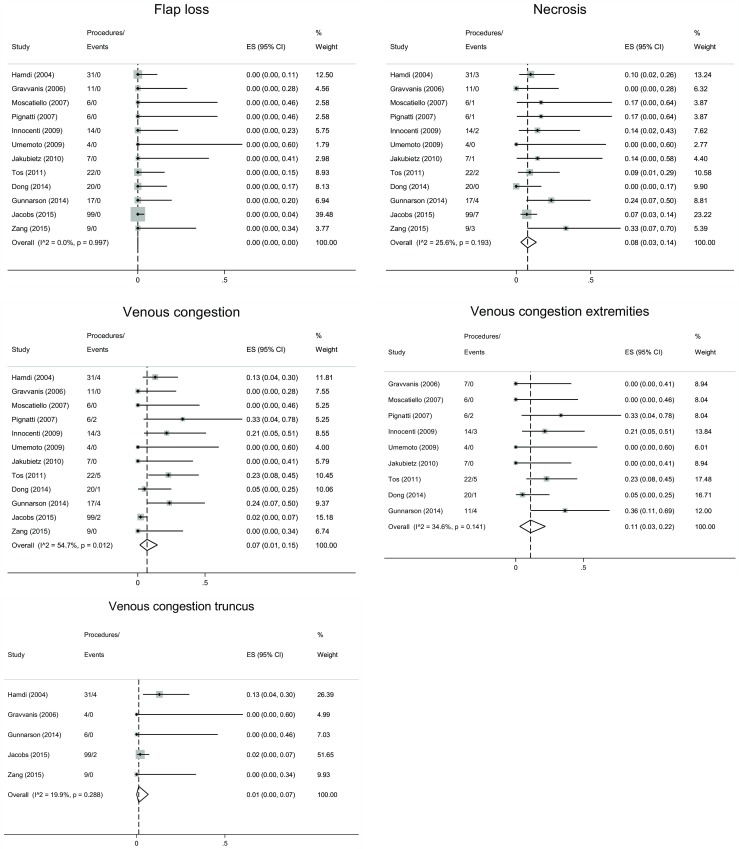
Meta-analysis for the effect size of complication rates: Flap loss (top left), necrosis (top right), venous congestion whole body (middle left), venous congestion extremities (middle right), and venous congestion truncus (bottom). Calculated for the random-effects model meta-analysis. *I*^2^: the percentage of total variation across studies which is due to heterogeneity

Surgical revision in general anesthesia was needed due to major complications in 21/252 (8%) cases. The re-operations were performed due to necrosis 16/252, venous congestion 2/252, hematoma 1/252, and epidermolysis 2/252 (Table 2). There were 36 cases of minor complications (14%) (Table 2). The most frequent was venous congestion 19/252 followed by tip necrosis 13/252, wound dehiscence 2/252, and other reasons 2/252. The meta-analysis yielded summarized complication rates of 0% flap loss, 8% any necrosis, 7% venous congestion throughout the whole body, 11% venous congestion in the extremities, and 1% venous congestion in truncus (Fig. 3).

## Discussion

Venous congestion was the most common complication in this series, which coincides with previous reports using pedicled perforator flaps for reconstruction [3, 20]. The risk of venous congestion was significantly higher in the lower extremities (11%) than in the torso (1%), as described previously (Fig. 3) [3]. We do not know the reason for the different complication rates between the torso and lower limbs. We speculate if it may be due to the following:

1. A difference in arterial flow and venous return
2. A greater vascular pressure in the extremities compared to the central body
3. A greater risk of twisting of the veins at the site of fascial penetration in the limbs
4. A need for a greater arc of rotation, which was close to 180° in many of the described flaps.

**Fig. 4:**
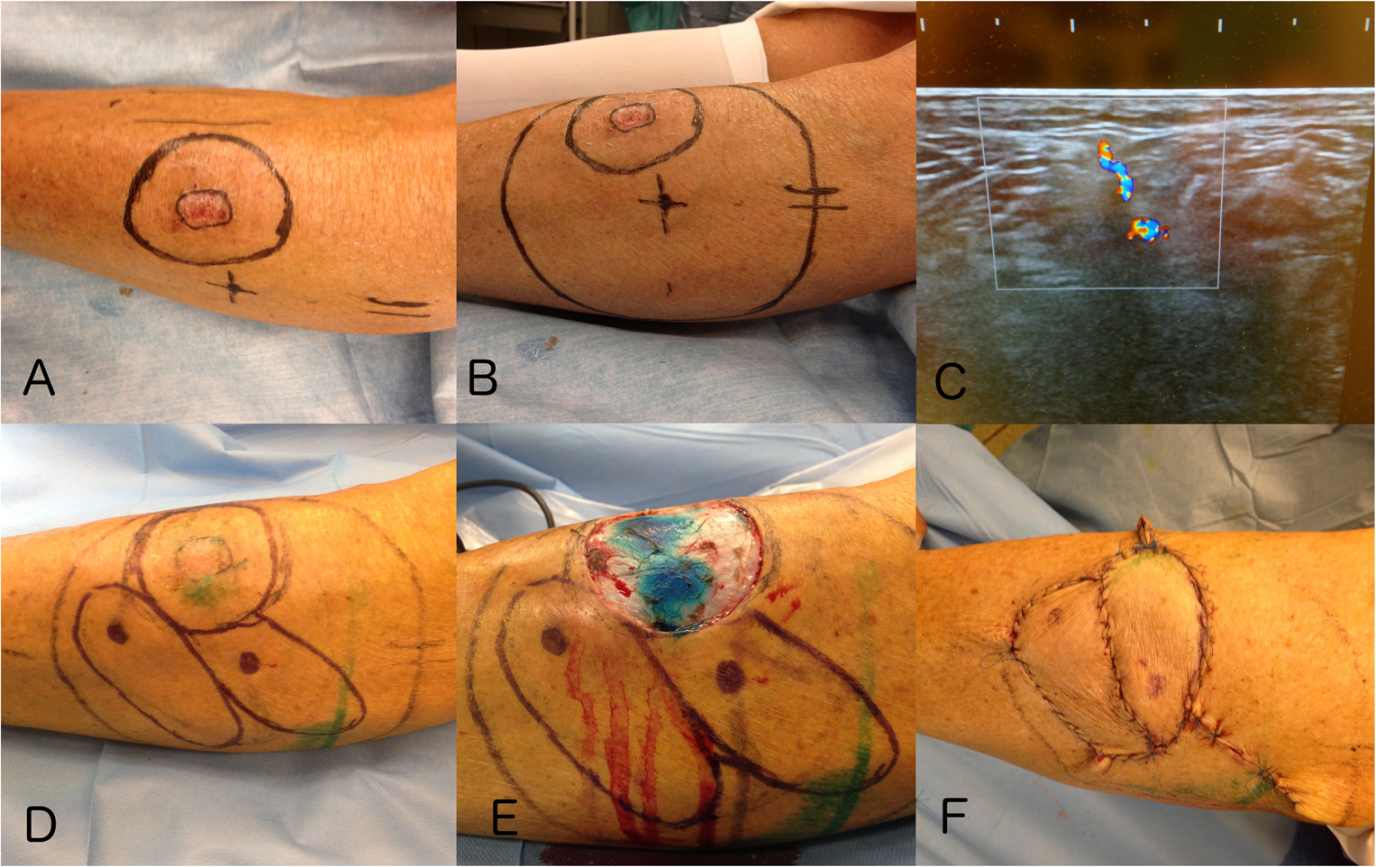
CDU targeted pedicled perforator flap reconstruction following excision of a malignant melanoma (MM) on the anterolateral lower limb. **a** Two-centimeter excision margin. **b** The largest perforator identified and the boundary of the possible donorsite marked by a circle. **c** The perforator identified by CDU. **d** Two perforators and two different flaps designs. **e** The MM excised. **f** The two perforator flaps transposed into the defect. One as a propeller

**Fig. 5:**
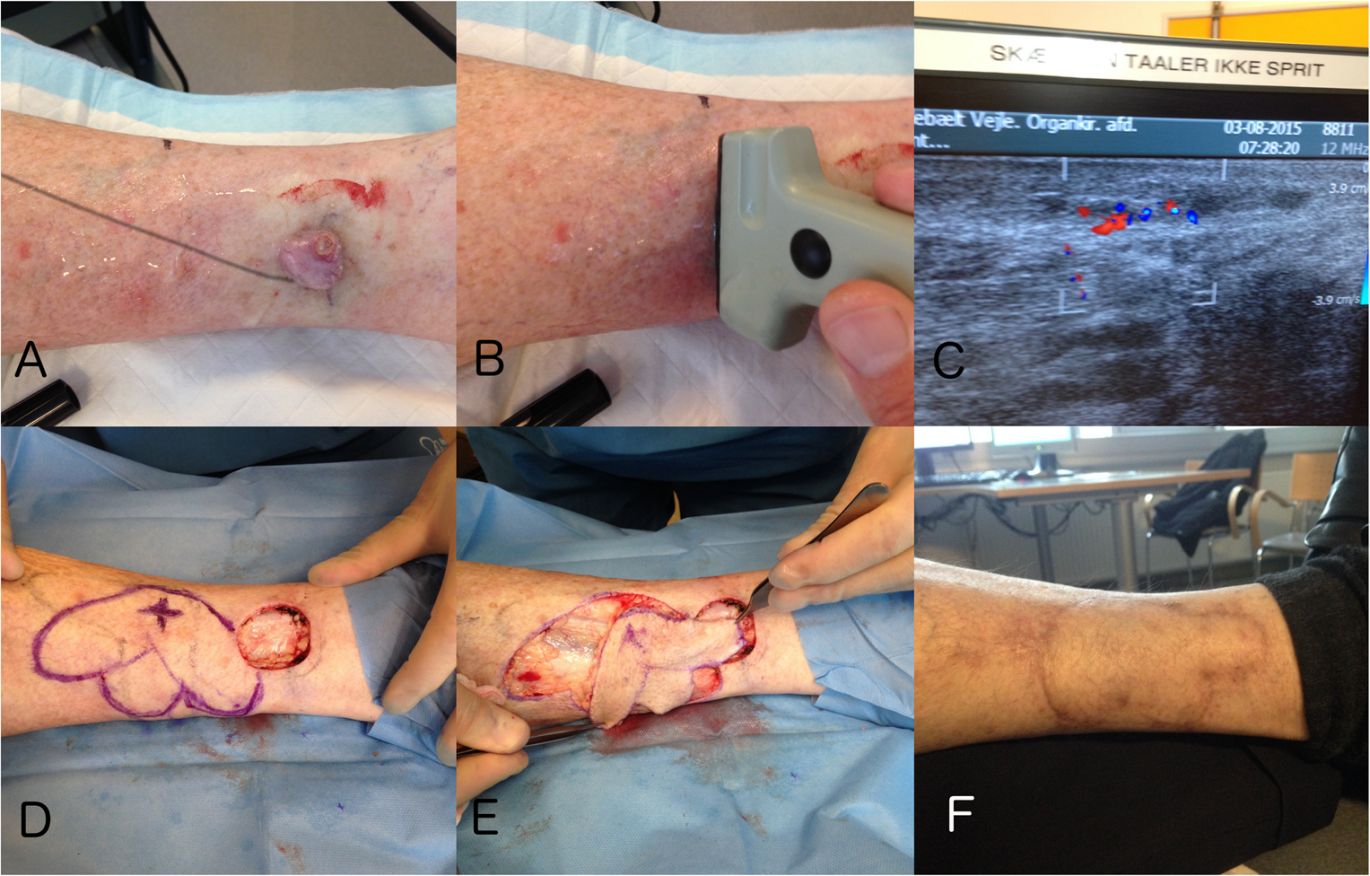
Trilobar flap used to limit the arc of rotation. **a** A carcinoma on the lower limb. **b**, **c** CDU identification of the largest accessible perforator adjacent to the defect **d** Marking of the perforator and a trilobar flap to minimize arc of rotation. **e** The flap propelled into the defect. **f** Long-term follow-up

The complication rate seems to increase along with an increasing arc of rotation compromising the vascular flow [3, 21]. It seems that the risk of complications is higher in areas where the amount of soft tissue is less abundant, like the distal part of the extremities. This translates to a shorter pedicle and restricted rotation, more prone to twisting and calls for greater dissection of the vessels or a different flap design for compensation (Fig. 5). This is where CDU may be helpful for designing the flap enabling precise planning based on the best-suited perforator, adjacent to the defect and with the best possible course through the subcutaneous tissue (Fig. 4). The use of bi-or multi-lobar flaps might be another solution which can reduce the arc of rotation from 180 to 90° or less [3] (Fig. 5).

The overall major complication rate was 8% in this review, which was lower than the 14% described by Andrea Sisti et al. in a literature review of 1315 propeller flaps without the use of CDU [20]. However, we cannot use these results to conclude that the use of CDU is associated with an overall lower complication rate although it may show a trend.

Interestingly, most of the included studies were small studies including 20 patients or less. Thus, the complication rates in this review, major 8% and minor 14%, have to be considered in the context of a learning curve setup. Better results should be expected once the learning curve is surpassed [11]. The summed major complication rate of the five smallest studies in this review was 16% compared to 8% overall, which is in accordance with Jiga et al. and Panse et al., who found that the overall outcome can be expected to improve while the complication rate decrease over time [22, 23].

The use of CDU for detection of perforators is observer dependent, which can be exemplified by two studies using CDU for detection of perforators for the harvest of the radial forearm flap. CDU was found to be extremely useful for detecting perforators for the radial forearm flap in one of these studies, yet the other study described difficulties using CDU to identify the perforators, because the signal from the radial artery shielded visualization of the perforators [18, 24]. It is therefore important to facilitate the correct use of CDU, which enables the surgeon to plan and design the pedicled perforator flap for reconstruction using the best available tissue adjacent to the defect, allowing for the least possible arc of rotation and least possible risk of complications, thus securing a successful reconstruction (Fig. 5).

The handheld Doppler is still an important tool for identification of perforators. However, CDU may have some advantages to the handheld Doppler. CDU has been found to be more precise and reliable than the handheld Doppler in the detection of perforating arteries of the anterolateral thigh [25, 26]. The CDU was able to visualize the perforator passage through the fascia, which the handheld Doppler could not. In another study comparing CDU and the handheld Doppler for the detection of the second dorsal metacarpal perforators, CDU identified more cutaneous perforators than the handheld Doppler. Furthermore, in some instances, the handheld Doppler mistook feeding vessels falsely as being perforators [27].

This systematic review revealed that the literature describing color Doppler ultrasonography used to identify perforators for pedicled perforator flap reconstruction of local defects is limited and the findings have some apparent limitations: first of all, the low level of evidence of the included studies. There was just one prospective study and 11 retrospective chart reviews. Secondly, several of the included studies were small case studies. On the other hand, the diversity of flaps and rate of successful reconstructions reported in this review seems to indicate that pedicled perforator flaps are a reliable alternative to other reconstructive options. Correct use of CDU enables the surgeon to target the perforator and plan a pedicled perforator flap for local reconstruction using the available tissue adjacent to the defect with the shortest possible arc of rotation or transposition to minimize the risk complications.

## Conclusion

The study revealed an 11 times greater risk of venous congestion when pedicled perforator flaps were raised in the extremities compared to the trunk. There is a learning curve associated with the identification of perforators using CDU. Furthermore, we need to adapt and individualize the flap design with regard to shape and size according to tissue availability and knowledge of perforator whereabouts and course. CDU can be used as a tool to identify perforators for pedicled perforator flap reconstruction; however, the literature related to this subject is limited.
